# Uneven geography of health opportunities among subsidized households: Illustrating healthcare accessibility and walkability for public rental housing in Seoul, Korea

**DOI:** 10.1371/journal.pone.0306743

**Published:** 2024-07-12

**Authors:** Junehyung Jeon, Ayoung Woo

**Affiliations:** Graduate School of Urban Studies, Hanyang University, Seoul, South Korea; Qatar University, QATAR

## Abstract

Planners and policymakers significantly consider providing suitable living environments for marginalized households, beyond creating affordable homes. Previous studies have explored various socioeconomic attributes of neighborhoods with public rental housing (PRH), particularly regarding education, job, and transportation opportunities; however, we have a limited understanding of health opportunities among such subsidized households. This study, therefore, explores the accessibility and spatial equity of emergency medical services (EMS) and primary health care (PHC) for PRH residents in Seoul, Korea. The findings show that neighborhoods with PRHs are associated with lower odds ratios for EMS and PHC accessibility. In particular, the relationships between the locations of PRHs and medical services accessibility in neighborhoods varied across the types of PRHs. While neighborhoods with large-scale PRHs are associated with lower PHC access, those with small-scale PRHs are associated with lower EMS access. In addition, our findings show that PRHs tend to be located in neighborhoods with lower walkability. These results may help in empirically determining the spatial accessibility of PHC and EMS, as well as neighborhood walkability, which may affect the health status of individuals in subsidized households.

## Introduction

Access to various amenities and resources in neighborhoods is essential for individuals to a better quality of life and socioeconomic mobility [1, 2]. However, access to socioeconomic opportunities varies depending on the neighborhood people live in [3–5]. Residents in wealthy and privileged areas may have better access to social and physical environments, such as higher quality education, higher wages, and safety. Conversely, people in deprived neighborhoods often attend relatively low-performing schools and experience high crime and unemployment rates and insufficient infrastructure [4]. Despite the known benefits of spatial opportunities and services, subsidized families tend to be concentrated in marginalized areas, dominated by poverty and insufficient amenities [2, 6, 7]. This uneven spatial pattern of subsidized housing may limit access to adequate socioeconomic opportunities and negatively affect the daily lives of low-income households [2]. Thus, besides providing affordable housing units to marginalized populations, it is vital for planners to ensure ‘suitable living environments’ in public rental housing (PRH) projects in both developing and developed countries.

Along with varied socioeconomic opportunities in neighborhoods, one of the essential components of the quality of neighborhoods is ease of access to health care. Health care opportunities are critical to individual health and social sustainability [8]. Health care access is associated with a direct link to individual quality of life, community health, and local economy [8, 9]. As the importance of quality health care is increasingly clear, health equity for fair distribution of health resources and facilities has become part of a primary agenda across public health experts and governments [8]. However, there is growing evidence that poor living environments surrounding PRH may worsen residents’ physical and mental health [10–12]. Spatial disparities in accessibility to medical services may negatively affect the public health outcomes and the ‘sustainability of daily routines’ for subsidized households. Previous studies have focused on the socioeconomic context of subsidized housing; however, little attention has been devoted to their geographical location and healthcare opportunities in neighborhoods. This study fills this gap by exploring whether PRH projects ensure ‘suitable living environments’ with regard to accessing health care opportunities.

This study addresses the following research question: Do neighborhoods where PRHs are located ensure access to healthcare opportunities for subsidized households? We examined the interrelationship of the locations of PRH and medical services accessibility in neighborhoods in Seoul, Korea. Our research employed a binary logistic regression method using a modified 2-Step Floating Catchment Area (2SFCA) measurement to identify whether PRH is placed in neighborhoods with adequate accessibility to primary health care (PHC) and emergency medical services (EMS). Furthermore, this study examined how healthcare attributes varied across the different PRH neighborhoods, classified by program characteristics such as methods of supplying, rental period, and eligibility. Our findings demonstrate a higher tendency for PRH to be sited in areas with limited medical accessibility. In particular, neighborhoods with large-scale PRH had limited access to EMS, while the small-scale projects had insufficient access to PHC. This study can help policymakers, planners, and public health practitioners better understand how healthcare opportunities are distributed across subsidized household neighborhoods, and can help tailor strategies to mitigate inequitable medical and healthcare accessibility.

## Literature review

### Uneven geography in subsidized housing

Socioeconomic opportunities are unevenly distributed across areas [3, 4, 13, 14]. This ‘uneven geography of opportunity’ exacerbates the differences among various socioeconomic benefits and amenities such as good quality housing, schools, workplaces, and safety from crime for residents in specific neighborhoods. Such differences have a profound influence on an individual’s quality of life and upward mobility. Furthermore, differences in access to socioeconomic opportunities may limit the wealth accumulation among disadvantaged populations [15]. Such situations may further hinder the disadvantaged populations from entering into desirable neighborhoods in the long run [15–17]. Given this vicious circle of poverty, the spatial location of housing, especially for disadvantaged populations, has become a paramount issue among planners and policymakers.

Given Seoul’s rapid population growth since the 1980s, the Korean government has provided affordable housing through various programs of PRH to ensure residential stability among marginalized populations [12, 18]. Large number of housing provisions have achieved quantitative results with a higher number of affordable units for marginalized families [18]. However, beyond ensuring quantitative achievements, planners and policymakers have increasingly agreed that subsidized housing projects need to provide suitable living environments with sufficient infrastructure, amenities, and landscape design to enhance the ‘sustainability of daily routines’ for marginalized residents [1]. Prior studies have empirically demonstrated that subsidized housing tends to be placed in marginalized areas, which generally correlate with higher poverty [19], higher crime rates [20], and lower Walk Score [2, 14]. Researchers have also highlighted that subsidized residents are geographically clustered in poor neighborhoods [1, 7]. The PRH policy, which has excessively focused on quantitative targets rather than quality of life, has further contributed to the uneven geography of subsidized households [18]. Despite the extensive literature examining various socioeconomic environments for subsidized households, to our knowledge, no studies have examined neighborhood environments supporting daily routines, especially in terms of accessibility to medical service and walkability, which are critical in maintaining and enhancing public health for subsidized families.

This study addresses the gap by empirically specifying the correlations between the locations of PRHs and neighborhood environments in terms of walkability and medical service accessibility. Specifically, this research examines different accessibility measures to consider the distinct characteristics of PHC and EMS. Furthermore, we elucidate walkable environmental characteristics based on the notion of 3Ds (i.e., density, diversity, and design). Our approaches may provide a better understanding of the importance of PRH locations and useful insights on how to ensure health opportunities for subsidized households by modifying neighborhood environments. Based on an extensive literature review on medical service accessibility and walkability, our study conceptualizes a framework for relationships between PRH locations and health opportunities and empirically investigates hypothesized relationships using the framework.

### Importance and measurement of health-related opportunities for subsidized households

Medical service opportunities are essential to maintaining and enhancing the public’s health and life quality [8, 9]. Therefore, planners and public health practitioners ensure medical accessibility by identifying the spatial distribution of medical resources (e.g., personnel and facilities) across neighborhoods [8]. Past literature is replete with studies describing how marginalized people have been afflicted with health problems and are spatially disadvantaged in health care opportunities [9, 21]. Compared with residents living in affluent areas, households in deprived areas often experience lower physical and psychological health outcomes and a higher risk of mortality [21]. Additionally, neighborhoods with low-income families have limited access to medical services, thereby worsening their health outcomes [11]. In particular, subsidized housing residents tend to experience poor mental health owing to social stigma and discrimination by neighbors [22]. The location and poor quality of PRH, with the prevalence of pests and rodents, result in greater exposure to criminal risks and poor mental health in subsidized households [11, 23]. Furthermore, the lack of amenities (e.g., shopping, healthy foods, and natural sites) around PRH and unsafe pedestrian conditions may hinder residents’ physical activity and management of obesity and chronic disease [10]. Despite the critical importance of maintaining health and better quality of life for subsidized housing residents, few studies have identified the opportunity of access to medical services in neighborhoods where such PRH is located.

Healthcare accessibility is often a criterion for a clear diagnosis of neighborhood healthcare quality [24]. Geographical proximity to all types of healthcare facilities has been universally employed to identify medical service accessibility in neighborhoods [12, 25–27]. However, we specify medical service accessibility using separate measures (i.e., density- and distance-based measures) because the PHC and EMS have varying characteristics including the supply volume and scale of facilities, frequency of visits, and travel mode for visiting [24, 26, 28]. PHC focuses on maintaining daily public health to prevent chronic and severe diseases [8, 9]. Compared with EMS, PHC has a higher visiting frequency owing to the relatively lower economic barrier and higher daily accessibility [28]. Spatial health equity for PHC opportunities among residents may be identified by the distribution of facilities according to supply scale and density by neighborhoods. Hence, this study employs density-based measures to account for the accessibility of PHCs across neighborhoods.

Conversely, EMS is part of a comprehensive medical system consisting of report registration and dispatch, pre-hospital care, patient transport, and definitive care. EMS is an essential medical service that saves lives in hazardous and urgent situations [29, 30]. Given that EMS is substantially affected by timely access to ambulances responsible for patient transfer, accurate measurement of ambulance travel and response time is critical to precisely measure the significance of such services in neighborhoods [29, 30]. Significantly, identifying the specific path and travel time based on dynamic traffic information may enhance the accuracy of accessibility estimation [30, 31]. However, prior literature has estimated the route to a health facility based only on simple Euclidean or network distances, and do not account for dynamic traffic information and travel time; these approaches can over- or under-estimate accessibility [27, 30]. Thus, beyond simple geographical proximity to EMS, this study fills this gap by employing a modified 2SFCA measurement based on a real-time navigation path application programming interface (API) to identify the accessibility of EMS across neighborhoods.

Along with healthcare accessibility in neighborhoods, a walkable environment is also critical for the enhancement of residents’ health. Walkable neighborhoods promote physical activity and may prevent an increased risk of obesity, type 2 diabetes, cardiovascular disease, and mental disorders [10, 32]. A multitude of health benefits have been attributed to walking, which does not incur costs and is an easily accessible physical daily activity [2]. Despite varying definitions of neighborhood walkability, planners have increasingly considered 3D elements—density (e.g., population density), diversity (e.g., land use mix), and design (e.g., street intersection and crosswalk)—to identify walkable environments in neighborhoods. Additionally, public transit is important in supporting daily trips, improving accessibility to various socioeconomic services further away, and is especially a dominant means of access for PHC [28]. Pedestrian-friendly environments are closely associated with carrying out daily requirements and maintaining the health of residents [10, 33, 34]. Despite the significance of walkable environments among subsidized residents, little research has been conducted on attributes concerning neighborhood walkability around PRH. Thus, this study specifies whether PRH helps subsidized households access to pedestrian-friendly environments. Furthermore, this research expands on the two types of PRH classified by methods of PRH supply, rental periods to explain how healthy living environments (i.e., PHC, EMS, and walkability) vary between large- and small-scale PRH.

## Methods

### Study area and description of Korea housing policy for low-income families

This study examined disparities in access to medical services and walkable environments according to neighborhoods with and without PRHs in Seoul, Korea. Seoul, the capital of South Korea, is one of the largest Asian cities, with an approximate population of 10 million residents [35].

**Fig 1:**
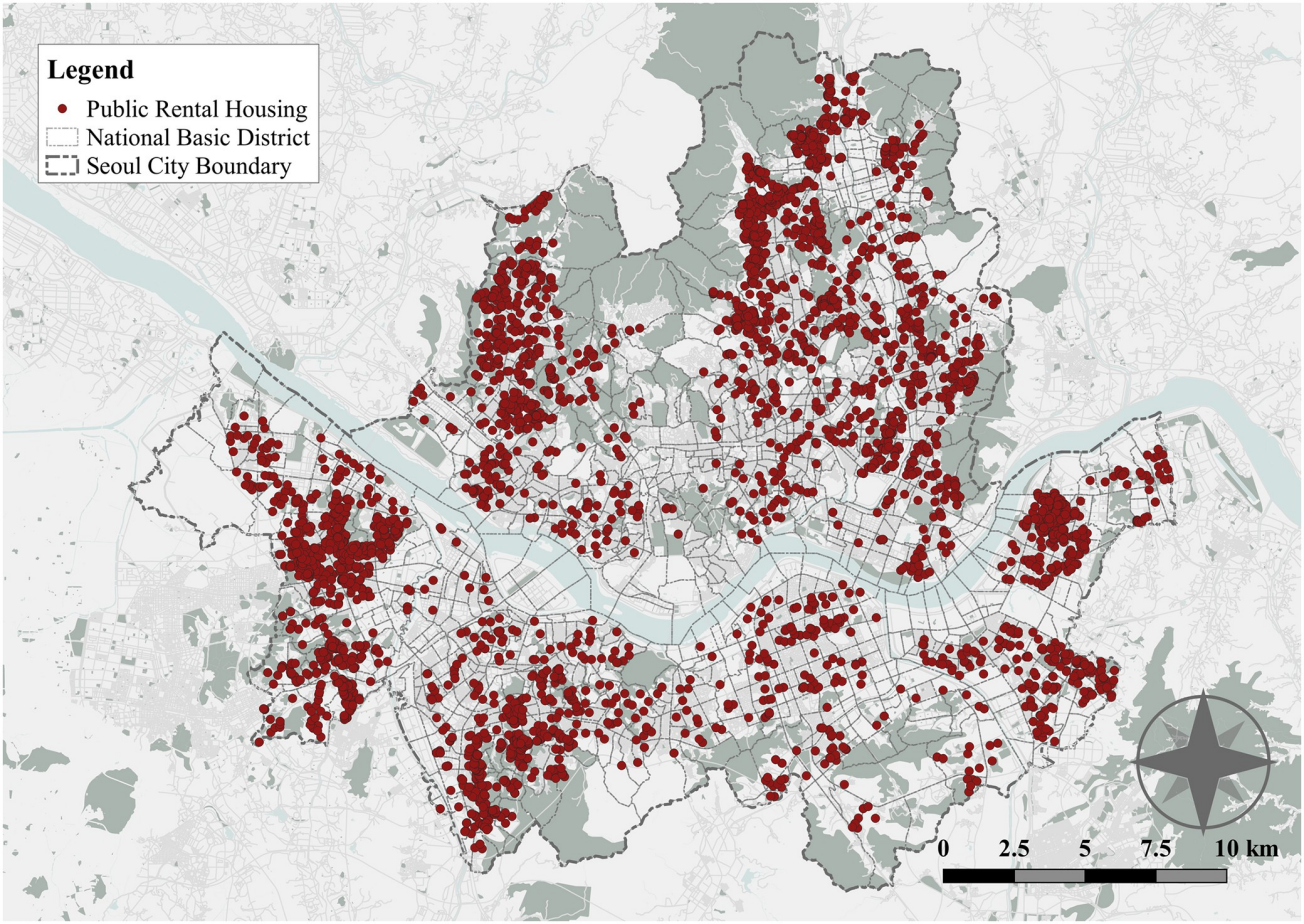
Spatial distribution of PRH in Seoul.

Fig 1 shows the spatial distribution of all PRH in 2019 Seoul. Although there are various types of PRH units in Korea, these units can be categorized into two types based on the leasing period, eligible income groups, and development methods: large-scale and small-scale PRH (Table 1). Large-scale PRH is classified as permanent public rental housing (PPRH), 50-year public rental housing (50 PRH), national public rental housing (NPRH), and redevelopment public rental housing (RPRH) [18]. This type of housing has been developed for extremely and very low-income households, ensuring a 30 to 50-year lease period, and is an effective method for providing affordable housing in specific sites intensively and largely.

**Table 1:** Status and classification of public rental housing programs.

Programs	Periods of Provision (year)	Qualification (avg. income)	Rental periods (maximum no. of years)	Methods of supplying in sites	Classification
**PPRH**	1989–1993	Less than 50% ^a^	50	Specific site	Large-scale
**50 PRH**	1993–1997	Less than 70%	50	Specific site	Large-scale
**RPRH**	1992–Present	50–70%	50	Specific site	Large-scale
**NPRH**	1998–Present	Less than 70%	30	Specific site	Large-scale
**PLPRH**	2003–Present	70–90%	6–20	Scattered-site	Small-scale
**SHIFT**	2007–Present	70–150%	20	Scattered-site	Small-scale
**Happy Housing**	2013–Present	Less than 100%	6–20	Scattered-site	Small-scale

Source: Data from Seoul Housing & Communities Corp. (SH) and Korea Land & Housing Corp. (LH)

^a^ Household income is below 50% of the average monthly income level of Seoul city workers.

In contrast, small-scale PRH includes shift housing (SHIFT), happy housing, and purchased and leased public rental housing (PLPRH). These have expanded their eligibility to low-income, young, and middle-income households, unlike large-scale PRH. However, the upper limit of the leasing period has decreased to less than 20 years [18]. Furthermore, PRH, categorized into small-scale, has deconcentrated across advantaged neighborhoods with small units rather than large-scale one as the alternative to address neighborhood resistance and provide suitable places to live for low-income families [1]. By distinguishing between large- and small-scale PRH, this study examines how medical services and neighborhood walkability vary across neighborhoods with different types of PRH, especially pondering which specific environments of each PRH improve or worsen health outcomes of subsidized residents.

### Measurement of the environmental attributes around public rental housing

This study used the National Basic District (NBD), akin to the U.S. Census Block Group, as the analysis unit referring to neighborhoods. NBD is a unit district created to provide statistical data based on roads, rivers, and mountain ranges in Korea [36]. The average population of NBDs in Seoul is approximately 6,700, and the average area is 0.43 square kilometers. Given that a half-mile distance is typically a walkable distance (i.e., 10-minute walking distance) [5, 14] and the PHC range is supposed to be within 10 minutes on foot [28], NBDs may be an apposite unit for measuring neighborhood medical services and physical environments. This study analyzed 1,377 out of 1,417 NBDs in 2019; we excluded 40 NBDs where residents did not live (e.g., temporarily relocated due to redevelopment and reconstruction) in Seoul.

**Fig 2:**
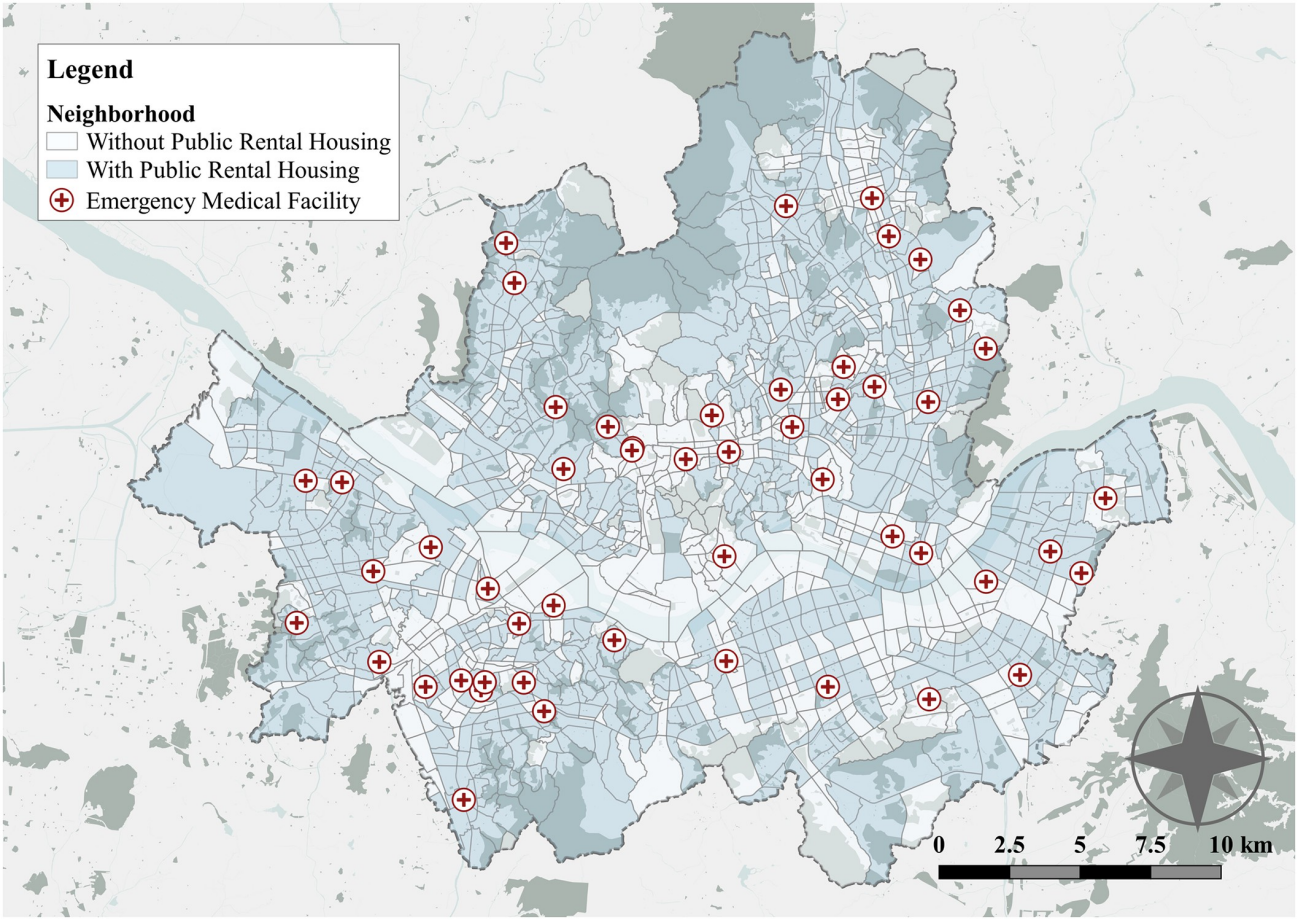
Emergency medical facilities for subsidized households in Seoul, Korea.

This study is particularly focused on ensuring EMS accessibility alongside PHC accessibility in PRH developments. To assess EMS accessibility for subsidized households, we identified the locations of fifty-one hospitals designated as EMS institutions in Seoul using data from the National Medical Center. Fig 2 shows that thirty-two out of the fifty-one EMS institutions in Seoul were mainly clustered in the southwest and northeast areas, whereas only four hospitals were contained in the northwest regions. In addition, this study also obtained data for the dispatch center from the National Fire Agency to consider the pre-hospital phase in the EMS system. Based on these data, our research investigated the disparity in EMS accessibility by neighborhoods with and without PRHs and clarified whether these differences varied across the types of PRH developments.

This study utilized various datasets to identify PRH neighborhoods’ medical service accessibility and environmental attributes that may affect residents’ health opportunities. Table 2 presents the description and the variable sources and measurement methods used in the analysis. We recognized the type and location of PRH in 2019, using data from the Korea Land and Housing Corporation (LH) and the Seoul Housing and Communities Corporation (SH). The ratio of NBDs where PRH was developed was 0.592. In terms of socio-demographic characteristics, we obtained annual median household income 2019 data from the Korea Credit Bureau (KCB) and identified land price information from the 2019 Seoul Metropolitan Government data. Apartments form the main housing type, accounting for approximately 50.1% of the entire housing units in Korea, and PRH is primarily provided in this form [37]. Therefore, this study included apartment variables from the 2019 census data of Statistics Korea to account for the location of PRH. Additionally, our study included the elderly population and single-person households to account for disadvantaged population attributes in neighborhoods.

**Table 2:** Descriptive statistics and measurements.

Variables	Measurements	Mean	S.D.	Min.	Max.	Source
**Dependent Variable**						
All public rental housing	Public rental housing in NBD (yes = 1, no = 0)	0.59	0.49	0	1	SH & LH (2021)
Large-scale PRH	Large-scale public rental housing in NBD (yes = 1, no = 0)	0.17	0.38	0	1	
Small-scale PRH	Small-scale public rental housing in NBD (yes = 1, no = 0)	0.49	0.50	0	1	
**Socio-demographic Characteristics**						
Elderly population	Percentage of people over 65 in NBD	15.23	5.05	1.20	80.73	Statistics Korea (2019)
Single-person households	Percentage of single-person households in NBD	33.75	16.54	3.43	89.29	
Apartment	Percentage of apartment housing in NBD	46.781	35.853	0	100	
Income	Median annual income (10,000 KRW ^a^) in NBD	3,823.72	892.75	2,499	11,401	KCB (2019) ^b^
Land price	Median land price (10,000 KRW ^a^) in NBD	495.71	382.77	17.070	4,815.5	Data Seoul (2019)
**Medical Services Accessibility**						
EMS Accessibility	No. of EMS beds per 10,000 population accessible in 30 min by vehicle (measured by the 2SFCA model)	1.503	1.349	0	7.280	SK API (2019)
PHC Accessibility	No. of PHC facilities ÷ total population in NBD (10 K)	47.72	233.45	0	5,666.67	KLID ^c^ (2019)
**Neighborhood Walkability: Transit Accessibility**						
Subway	No. of subway station entrances ÷ total street length in NBD (km)	0.22	0.55	0	13.80	Data Seoul (2019)
Transfer	Subway transfer station (yes = 1, no = 0)	0.140	0.347	0	1	
Bus	No. of bus stops weighted by no. of lines ÷ total street length in NBD (km)	4.31	4.61	0	101.13	
**Neighborhood Walkability: Built Environments**						
Population density	Total population (1,000) ÷ NBD area (km2)	23.39	13.25	0.03	68.51	Statistics Korea (2019)
3-leg intersection	No. of 3-leg intersections ÷ total street length in NBD (km)	2.11	2.23	0	18.02	KTDB ^d^ (2019)
4-or-more-leg intersection	No. of 4-or-more-leg intersections ÷ total street length in NBD (km)	0.71	0.93	0	8.39	
Mixed land use	Entropy Index (0–1) ^f^	0.62	0.19	0	1	MOLIT ^e^ (2019)
Crosswalk	No. of crosswalks ÷ total street length in NBD (km)	5.09	3.18	0	19.61	Data Seoul (2019)
**Locational Characteristics**						
Geographic Coordinates	X coordinates (normalized by the distance to CBD) of the centroid of NBD			-0.19	0.20	Statistics Korea (2019)
	Y coordinates (normalized by the distance to CBD) of the centroid of NBD			-0.13	0.12	

^a^ About 9 USD

^b^ Korean Credit Bureau

^c^ Korea Local Information Research & Development Institute

^d^ Korea Transportation Data Base

^f^ Entropy Index is calculated with mixed land use = −∑_*k*_ (*p*_k_ ln *p*_*k*_) ÷ ln *N*, where pk = proportion of total floor area by measured land use k and N = total number of land use types in NBD. This index varies across 0 to 1 and means that if the index is close to 1, the census block is composed of greater land use diversity [38].

^e^ Ministry of Land, Infrastructure and Transport

In terms of walkable environments that encourage subsidized household health, we identified public transportation accessibility and built environmental features in neighborhoods. This study used the 2019 subway entrance data from the Ministry of Public Administration and Security and the 2019 bus-stop location data from the Seoul Metropolitan Government to identify public transit accessibility. The accessibility to public transportation was measured by the density dividing the number of subway entrances and bus stops located in NBD by the total street length. This study, particularly, accounted for the number of lines at each subway and bus station to specify the magnitudes of the impact of transfer station. A dummy variable was included for subway transfers, and the bus station was summated using all lines passing the stops in the density calculation.

Built environments for neighborhood walkability were identified by population density, intersection density, and mixed land use, as suggested by the 3Ds that are widely applied in active transit research [14, 38, 39]. We included the total population density of the neighborhood using a census in 2019. This study also utilized the intersection data provided by the Korea Transport Database to explore intersection density; in particular, we distinguished three-leg intersections and four-or-more-leg intersections. We also included crosswalk density related to pedestrian safety. Finally, this study included mixed land use in the neighborhood expressed as an entropy index.

Location characteristics include longitudinal and latitudinal measures of each NBD centroid normalized by the distance to the CBD to explain the spatial autocorrelation issue [14].

### Spatial accessibility of medical services

This study examined the differences in the spatial accessibility of medical services between neighborhoods with and without PRH. We assessed the PHC using density-based measures. There were 16,614 PHCs in Seoul, based on the 2019 local data [40].

Such density-based measures may be an intuitive and relatively simple way to measure the spatial accessibility of PHCs in neighborhoods [9, 41]. These density-based measures are appropriate where service volume and routine visits are essential, such as PHC or non-urgent access requirements. However, density-based measures for EMS have several limitations, particularly when the supply is limited, and the scale and distance of medical facilities need to be examined closely [24]. Density-based measures do not consider the actual travel distances for EMS facilities among neighborhoods. Furthermore, there is a constraint in estimating accessibility if EMS facilities are located outside a geopolitical boundary (e.g., NBD), but the actual distance from patients is close [42]. Hence, to overcome these gaps in density measurements, the 2SFCA has been employed in public health and transportation-related studies [24, 25, 42, 43].

The 2SFCA method measures potential accessibility by assessing areas of service provision for different capacities among medical facilities and the range of residents’ trips [42]. Thus, the 2SFCA is a fitting measurement for EMS wherein service scale and travel distance are emphasized more than volume, given the interrelationship of the medical institution’s service capacity and residents’ travel patterns in a neighborhood [9, 24].

The conventional 2SFCA estimates accessibility in two steps:

$$R_{j}=\frac{s_{j}}{\sum_{k\in \{d_{jk}\leq d_{0}}p_{k}}$$ (1)

$$A_{i}=\sum_{k\in \{d_{ik}\leq d_{0}}R_{j}.$$ (2)

The first step calculates the ratio (*R*_*j*_) of medical resources (i.e., available numbers of doctors or beds) (*S*_*j*_) to the total population (*P*_*k*_) within a threshold distance (*d*_*0*_), set based on each medical facility location *j* (Eq 1). The second step estimates healthcare accessibility (*A*_*i*_) in neighborhoods by the summation of all ratios (*R*_*j*_) of accessible medical resources within a threshold distance based on the centroid *i* of each area (Eq 2). The threshold in the formula refers to the maximum distance (or time) that residents may cover to access medical facilities from the neighborhood center. Although the threshold varies across researchers for each national and regional medical system, the Korean Ministry of Health and Welfare has identified a threshold distance to the Golden Hour, 30 minutes [44].

**Fig 3:**
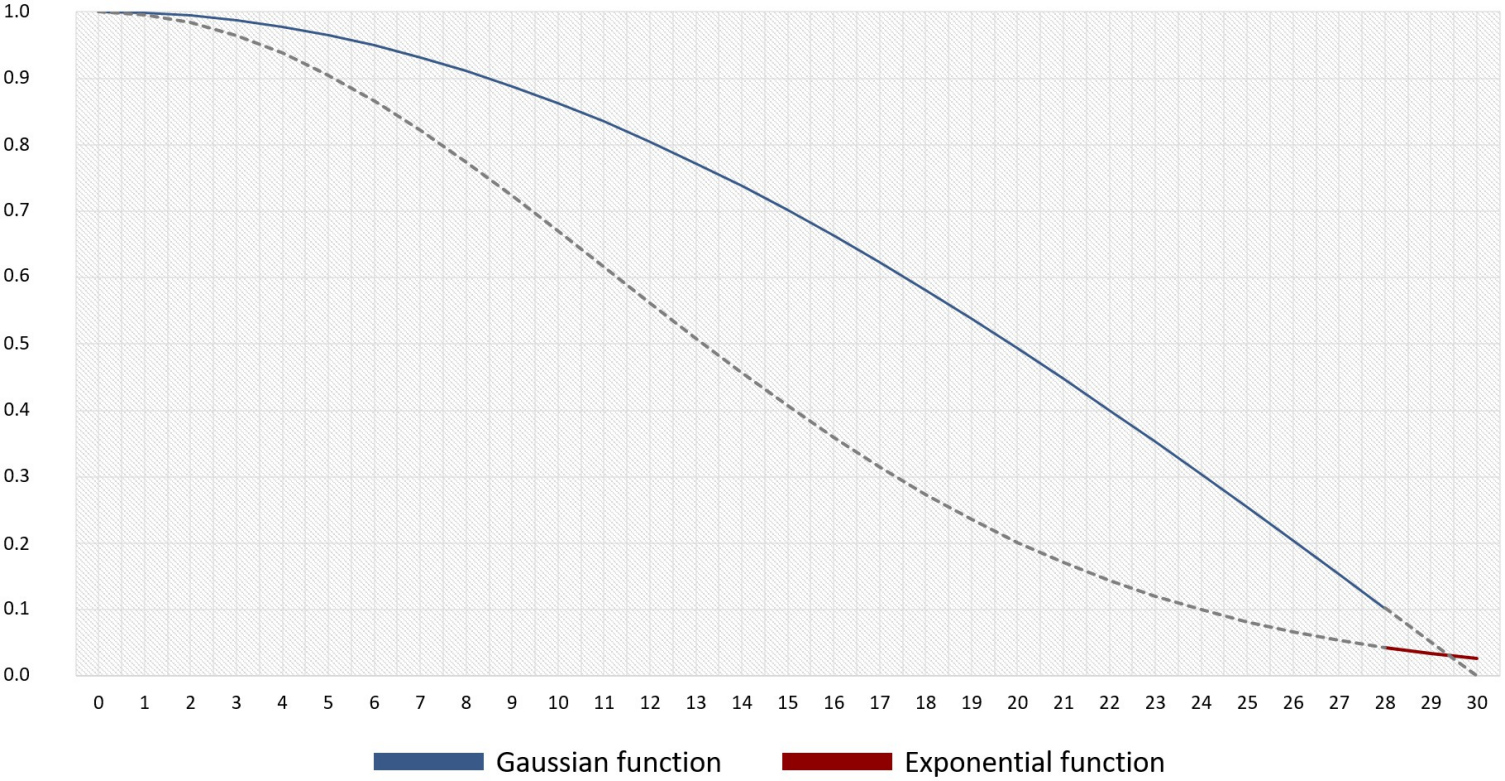
Distance decay with both Gaussian and exponential functions in the study.

However, the conventional 2SFCA model has attracted criticism because it assumes equal access within a catchment regardless of differences in travel distance. Hence, we used distance-decay functions, especially the combination of Gaussian [45] and exponential [26] functions, to specify the travel distance for EMS facilities in neighborhoods. Specifically, we applied the Gaussian function up to the average emergency transfer time in Seoul, and subsequently referred to the exponential function with a relatively lower weight than the Gaussian function to the Golden Hour, 30 minutes (Fig 3).

The Golden Hour is critical for patient survival. It is defined as the time window for trauma patients to receive definitive care for life, as well as the reference time for identifying medically vulnerable areas in Korea [44]. Distance decay becomes more critical as it approaches the Golden Hour because it is associated with the patient’s life and death, compared with before the average transfer time. Given that previous studies indicate that a patient’s symptoms and mortality may worsen as transport time increases [46, 47], we consider that applying a lower distance-decay function value is reasonable as the EMS reference time approaches the Golden Hour (30 minutes). Therefore, this study applied an exponential function afterward, adopting the Gaussian function to the average transfer time until the threshold distance. The average transfer time in Seoul was 28.3 minutes [48] and the threshold time was set to 30 minutes [44] to estimate the weight in the 2SFCA model.

Hence, our modified 2SFCA formula incorporates these critical factors more accurately, as follows: *R*_*j*_ is the ratio of bed *j* to the total population of basic district units accessible in 30 minutes (Eq 3). Next, the medical service accessibility *A*_*j*_ is investigated by summating the bed supply rate *(R*_*j*_*)* in the accessible emergency medical centers in the threshold time from each NBD centroid *i* (Eq 4). The Gaussian function is only applied within the average transfer time, and later, the exponential function has an effect until the threshold time (Eq 5).


$$R_{j}=\frac{S_{j}}{\sum_{k\in \{t_{jk}\leq t_{0}}P_{k}W\left(t_{jk}\right)}$$ (3)

$$A_{i}=\sum_{j\in \{t_{ij}\leq t_{0}}R_{j}W\left(t_{ij}\right)$$ (4)

Wtij=e-12×tijt02-e-121-e-12tij≤28.3e-0.9×tijω2,ω=tmax228.3≤tij≤30 (5)

However, there are further limitations to previous research employing the 2SFCA model: lack of consideration of 1) real-time travel distance and 2) other time factors to identify the threshold distance. First, many previous studies have utilized the Euclidian or network distance to set the geographical impedance [43]. This approach may be problematic because it only considers ideal accessibility and does not include real-time road information such as traffic patterns, traffic jams, and weather conditions. Thus, we estimated the travel time from the centroid of the NBD to emergency medical institutions using a real-time navigation route approach based on the SK open API. Criteria establishment is essential to obtain real-time information using API because arrival time may vary owing to traffic volume changes dependent on time and season. According to the 2018 Transferred Patients Statistical Yearbook, a higher number of patient transfers occurred in August, September, and December, concentrated mainly between 9 am and 10 am. Thus, we obtained data from December 2019 to January 2020, between 9 am and 10 am, considering the above.

Second, the spatial accessibility of EMS should consider the time for the entire transfer phase to EMS facilities to identify the threshold time (i.e., Golden Hour): activation, response, on-screen, and transport intervals. Beyond the time needed to transfer patients to EMS facilities (transport interval), other critically important time factors are the additional steps in the EMS that paramedics prepare before dispatch (activation interval), move to the emergency (response interval), and required treatment of patients after on-site arrival, such as triage (on-screen interval) [29]. However, prior studies have only focused on the transport interval, especially in terms of the distance between neighborhoods and EMS institutions, to account for the threshold distance in the modified 2SFCA model [30]. Hence, we included the response time, which is the time taken by each ambulance to travel from the actual starting point (i.e., dispatch centers) to the centroid of the neighborhood. This study also included the average time of dispatch preparation and site treatment time recorded in the first aid log for four years from January 1, 2011, to December 31, 2014, referencing to the criteria of previous research [29, 48]. The average time for activation and on-screen intervals were 1.68 and 7.98 minutes, respectively. Hence, this study estimated the accessibility of EMS by adding 9.66 minutes to the measured two-path travel time.

**Fig 4:**
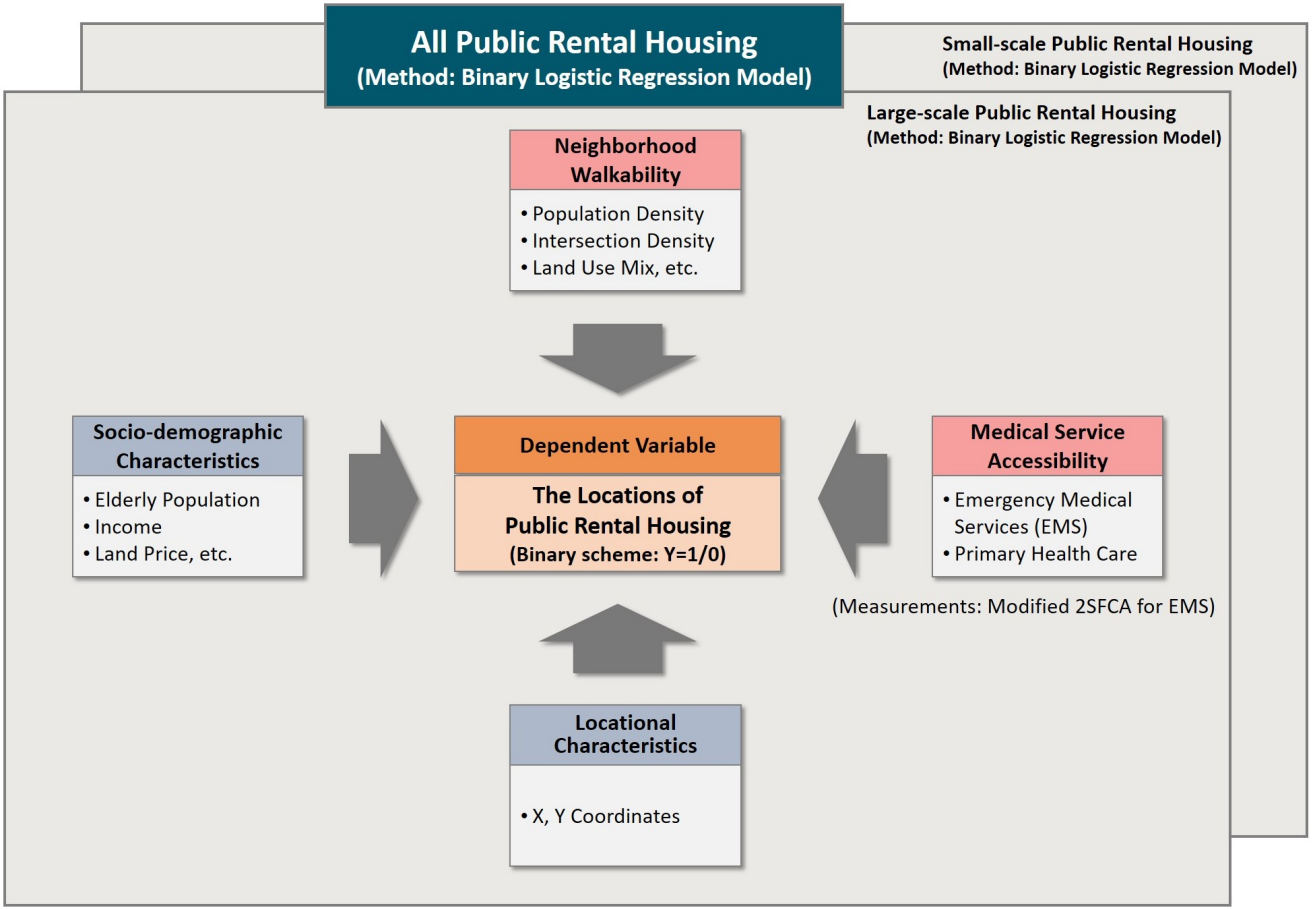
Conceptual model.

Our research conceptualized a framework to test the hypothesized relationship between PRH locations and health opportunities after accounting for socio-demographic and environmental determinants of previous studies on PRH locations (Fig 4). Using density-based and 2SFCA measurements, this study employed a binary logistic regression to explore health service accessibility and walkable environmental attributes of PRH neighborhoods. The dependent variable was whether PRH is located in a neighborhood. This study included socio-demographic characteristics, medical service accessibility, and differences in the physical environment to test our hypotheses. Additionally, we hypothesized that the relationships between PRH locations and health opportunities in neighborhoods may vary across different types of PRHs. Therefore, we stratified various PRH programs into large- and small-scale PRH based on rental periods and the methods of supplying in site. Using separate binary logistic regression models for large- and small-scale PRH, we examined the heterogeneous relationships between each PRH location and health opportunities in neighborhoods.

## Results

Table 3 shows the binary logistic regression results for all PRH in Seoul.

**Table 3:** Logistic regression results for overall public rental housing.

Variables	Coef.	OR (Odds Ratio)	*z*	*P*
**Socio-demographic Characteristics**				
Elderly population (%)	-0.015	0.985	-1.03	0.304
Single-person household (%)	-0.025***	0.975	-4.34	0.000
Apartment ratio (%)	0.004	1.003	1.31	0.191
Income	-0.044***	0.957	-3.67	0.000
Land price	-0.039	0.962	-1.22	0.223
**Medical Services Accessibility**				
EMS accessibility	-0.118**	0.888	-2.46	0.014
PHC accessibility	-0.002	0.998	-1.41	0.160
**Neighborhood Walkability: Transit Accessibility**				
Subway	-0.56 ***	0.569	-2.75	0.006
Transfer	-0.128	0.880	-0.64	0.520
Bus	-0.038*	0.962	-1.79	0.073
**Neighborhood Walkability: Walkable Environments**				
Population density	0.002***	1.002	4.20	0.000
3-leg intersection	-0.139***	0.870	-3.42	0.001
4-or-more-leg intersection	-0.260***	0.770	-2.27	0.002
Mixed land use	0.244	1.276	0.67	0.501
Crosswalk	-0.005	0.995	-0.16	0.875
**Locational Characteristics**				
Geographic Coordinates (X and Y)	Included			
N	1,377			
Prob > chi^2^	0.000			
Cox-Snell R^2^	0.190			
Nagelkerke R^2^	0.256			

*** *p* < 0.01;

** *p* < 0.05;

* *p* < 0.10

Our findings suggest that the relationships between the spatial location of PRH and socio-demographic characteristics were consistent with those of previous researches. The percentage of single-person households was negatively associated with the locations of overall PRH (OR = 0.975, *p* < 0.01); as single-person households in the neighborhood increased by 1%, the odds of PRH being located there decreased by 2.5%. Additionally, the annual median income of a household had a negative correlation with areas where PRH was located. In the binominal logistic model for overall PRH, the variables of the elderly population, apartments, and land prices were not statistically significant.

In terms of overall PRH location, PHC accessibility was not found to be significant. However, EMS accessibility had a negative relationship with overall PRH location (OR = 0.888, *p* < 0.05), signifying that PRH was more likely to be developed in neighborhoods with low access to EMS.

For transit services in neighborhood walkability, the subway density feature was negatively correlated with the location of PRH (OR = 0.569, *p* < 0.01); hence, it can be inferred that PRH was primarily sited in the neighborhood with limited access to subway stations. In addition, the odds ratio (OR) for bus-stop density was 0.962. However, this result was only statistically significant at the 10% level (*p* = 0.073). Regarding the associations between neighborhoods where PRH was located and walkable environments, population density had a positive significance with PRH in Seoul. In contrast, 3-leg intersection and 4-or-more leg intersection densities had negative relationships with overall PRH neighborhoods, indicating the tendency for most PRH to be developed in communities designed as low 3-way (OR = 0.870, *p* < 0.01) and 4-way intersection densities (OR = 0.770, *p* < 0.01).

An additional analysis was conducted to elucidate the differences in medical services and walkable environments between large- and small-scale PRH. Table 4 reveals the distinct differences between large- and small-scale PRH.

**Table 4:** Logistic regression results for large-scale and small-scale public rental housing.

Variable	Large-scale public rental housing				Small-scale public rental housing			
	Coef.	OR	*z*	*p*	Coef.	OR	*z*	*P*
**Socio-demographic Characteristics**								
Elderly populations	0.084***	1.087	3.94	0.000	-0.088***	0.915	-5.20	0.000
Single-person households	0.036***	1.036	4.01	0.000	-0.047***	0.954	-7.61	0.000
Apartment ratio	0.052***	1.054	11.37	0.000	-0.017***	0.983	-5.92	0.000
Income	-0.080***	0.923	-3.81	0.000	-3.22e-04***	9.997e-01	-2.63	0.008
Land price	-0.120**	0.887	-2.16	0.031	-4.130e-04	9.9996e-01	-0.13	0.900
**Medical Services Accessibility**								
EMS accessibility	-0.055	0.946	-0.74	0.462	-0.132***	0.876	-2.73	0.006
PHC accessibility	-0.012**	0.988	-2.29	0.022	-0.001	9.987e-01	-1.12	0.262
**Neighborhood Walkability: Transit Accessibility**								
Subway	-0.643**	0.526	-2.23	0.025	-0.618**	0.539	-2.59	0.010
Transfer	0.525*	1.690	1.78	0.075	-0.167	0.847	-0.80	0.422
Bus	-0.018	0.983	-0.70	0.485	-0.015	0.985	-0.64	0.522
**Neighborhood Walkability: Walkable Environments**								
Population density	0.015**	1.015	2.21	0.027	4.33e-06	1.000004	0.81	0.418
3-leg intersection	-0.034	0.966	-0.69	0.491	-0.144***	0.866	-3.19	0.001
4-or-more-leg intersection	-0.216**	0.806	-2.10	0.036	-0.290**	0.748	-3.05	0.002
Mixed land use	-0.575	0.563	-1.16	0.248	0.396	1.486	1.08	0.280
Crosswalk	0.116***	1.122	3.10	0.002	-0.077**	0.925	-2.48	0.013
**Locational Characteristics**								
Geographic Coordinates	Included				Included			
N	1,377				1,377			
Prob > chi^2^	0.000				0.000			
Cox-Snell R^2^	0.238				0.221			
Nagelkerke R^2^	0.395				0.295			

*** *p* < 0.01;

** *p* < 0.05;

* *p* < 0.10

In terms of socio-demographic characteristics, the annual median household income was negatively related to both PRH programs. However, other socio-demographic variables showed contrasting results between large- and small-scale PRH. Land price features were significantly and negatively associated with large-scale PRH location but were not statistically significant with the small-scale PRH locations. Additionally, the percentages of the elderly population, single-person households, and apartment types had a significant positive correlation for large-scale PRH but a negative correlation for small-scale PRH, which reveals contrasting results.

The results for medical service accessibility, our paramount concern, varied significantly according to different types of PRH locations. Neighborhoods with large-scale PRH showed a negative association with PHC access, indicating that such housing was concentrated in areas with low PHC access. For EMS accessibility, the relationship with large-scale PRH was not significant. Conversely, the finding for EMS was negatively associated with neighborhoods with small-scale PRH; thus, such households were distributed in areas with vulnerable EMS accessibility.

Regarding neighborhood walkability, subway density had a negative relationship with both locations of large- and small-scale PRH. The population density showed a positive correlation with large-scale PRH locations. Conversely, there was no statistically significant difference between small-scale PRH locations and population density. The variable signifying varied correlations across programs was the crosswalk density. Generally, large-scale PRH was associated with neighborhoods with higher crosswalk density, while small-scale housing was related to lower crosswalk density. Finally, three-leg intersection density was negatively correlated only with neighborhoods surrounding small-scale PRH. In contrast, there were statistically and negatively significant differences between four-or-more-leg intersection densities and large-scale and small-scale PRH.

## Discussion and conclusion

Subsidized households are often exposed to deteriorating health risks, given their socioeconomic status and the built environments around them [11, 21]. However, despite the great concern regarding potential physical and mental health problems for subsidized families, insufficient attention has been paid to the empirical correlation between the location of subsidized families and the spatial accessibility of key indicators (i.e., medical services) representing quality of life. Furthermore, few studies have clearly distinguished the consequences of insufficient access to PHC and EMS. Along with the estimation of spatial accessibility according to disparate medical services, this study also illuminated critical components of health equity for marginalized populations by identifying the relationship between PRH location and walkable environments that may affect individual public health.

This study demonstrates that PRH tends to be situated in areas with low single-person households and annual median household income. In particular, these socioeconomic characteristics of PRH showed different results according to the duration of these programs—large-scale or small-scale. Large-scale PRH was positively related to elderly and single-family households; conversely, small-scale PRH was negatively associated with these populations. There are also conflicting land price results for different PRH types. Large-scale developments often require large pieces of land within a limited budget. Thus, large-scale PRH was mainly developed by a massive apartment complex, and had positive correlations with neighboring apartment ratios and low-cost sites. In contrast, the results for land price and apartment ratio in small-scale PRH, or a scattered-site housing, were negatively associated or not statistically significant.

The findings of this study also showed that accessibility to medical services varied among neighborhoods with large-scale and small-scale PRH projects. This study found that large-scale PRH tends to be sited in areas with limited access to PHCs. This spatial pattern is based on the fact that large-scale homes do not guarantee residents health benefits in combatting chronic diseases. In contrast, small-scale homes are generally developed in neighborhoods with lower EMS access. This result implies that small-scale PRH has limited access to timely medical care, which leads to a higher risk of fatalities among residents in emergency events.

Therefore, more targeted approaches should be considered to improve medical service accessibility in large-scale and small-scale PRH in Seoul. Municipal governments should consider installing additional community-based public health facilities to enhance PHC accessibility in areas where large-scale PRH is situated. As previous studies highlight that PHC centers are often concentrated in wealthy and higher social status neighborhoods [25], targeted public investments are essential to expand PHC around PRH locations. Indirect solutions can be efficient in retrofitting EMS accessibility owing to constraints such as the demand for professional health personnel and the scale of facilities. For instance, establishing an effective patient transportation system can be critical in improving EMS accessibility for neighborhoods with small-scale PRH. Given the positive correlations between expeditious EMS onset time and reduction in delayed treatment and mortality rates [46, 47, 49], planners and policymakers should ensure efficient deployment and supply of the dispatch centers to reduce the travel time to sites and improve the availability of patient transport. Furthermore, reconsideration of the present supply strategy may be necessary to address low EMS accessibility for small-scale PRH. This type of PRH is designed to secure housing stocks by purchasing or leasing existing homes citywide through applications from private homeowners [50]. Although a committee consisting of planners and architects screens candidate buildings based on guidelines for guaranteeing minimum housing standards during the process, the requirements primarily include dwelling size, conditions, and price. Hence, if buildings sited in EMS-vulnerable areas have no quality issues, housing authorities are likely to purchase or lease such buildings to achieve the goal of housing stocks. Therefore, the introduction of revised guidelines filtering whether living environments surrounding candidate homes provide medical accessibility may be helpful in providing better EMS accessibility for small-scale residents.

Along with achieving medical equity, retrofitting walkable environments around PRH is critical for the sustainability of daily routines. Despite this need, the empirical results of this study raise concerns about pedestrian safety and community public health owing to the low walkability found across PRH neighborhoods. As the block length increases and intersection density decreases, residents’ destination accessibility is hindered, with the increased risk of vehicle accidents [5, 51]. In addition, our findings revealed that PRHs tend to be developed in neighborhoods with insufficient transportation infrastructure. Literature on travel behavior has demonstrated that people living in communities with lower transit accessibility are more likely to choose vehicle trips for commuting and shopping [52–54]. Consequently, PRH neighborhoods with lower subway accessibility may generate more traffic volume, thereby exposing subsidized low-income families to a greater risk of car accidents [5, 51]. In particular, small-scale PRH residents may face severe injuries or fatal accidents caused by conflicts with automobiles while walking due to the low density of crosswalks that help drivers’ awareness and reduce vehicle speed [14, 51]. Despite these hazardous situations, low EMS accessibility in small-scale PRH could allow restricted surgical protection for low-income households. Therefore, transportation experts and housing authority officers should consider increasing crosswalks around small-scale PRH to reduce potential accidents and enhance neighborhood walkability. Furthermore, neighborhoods with large-scale PRH were found to have regions with higher elderly population ratios. Elderly populations generally tend to visit PHCs by public transit or on foot, but the lack of crosswalks and their decreased physical and perceptual abilities expose them to a greater risk of death and injuries from accidents. Therefore, traffic engineers should reduce potential accidents involving the elderly by designating additional speed restriction zones and installing traffic calming devices around large-scale PRH.

Subsidized households suffer from poor socioeconomic conditions, which often result in the deterioration of physical and psychological health. The findings of this study indicate that PRH neighborhoods are correlated with uneven accessibility to medical services. To ensure medical welfare for marginalized populations, housing authorities and policymakers should develop and adopt revised supply process guidelines that consider and evaluate whether the living environments around PRH can provide appropriate healthcare accessibility. Furthermore, the program requirements should be reinforced so that PRH is located in areas that ensure sufficient public transportation infrastructure and walkability. Even when medical services are provided, PRH units can be developed in impoverished neighborhoods with unfavorable access to walkable environments owing to various constraints, such as higher land prices, limitations of development costs, and NIMBY (Not In My Back Yard) attitudes. In particular, NIMBY attitudes from advantaged neighborhood residents are critical barriers preventing PRH developments [7, 55, 56]. Residents living in advantaged neighborhoods have vehemently opposed PRHs, characterizing them as undesirable facilities [1, 55, 56]. Under the influence of this negative community resistance, PRH locations have a tendency to be placed in disadvantaged neighborhoods with lower access to socioeconomic, environmental, and medical services opportunities [1, 14, 19]. However, at a minimum, the best alternative approaches and strategies should be planned to ameliorate conditions for the nation’s disadvantaged populations. To revamp existing housing programs and public health policies, stakeholders should facilitate collaboration amongst policymakers, planners, public health professionals, and housing and traffic experts. Effective planning methods must be developed and implemented to enhance accessibility to medical services and neighborhood walkability in PRH.

## Supporting information

S1 Data Analytical data set for the study. (DOCX)
