# Supplementary material for: Uneven geography of health opportunities among subsidized households: Illustrating healthcare accessibility and walkability for public rental housing in Seoul, Korea
Source: PLoS One. 2024 Jul 12;19(7):e0306743. doi: 10.1371/journal.pone.0306743 (PMC11244778; doi:10.1371/journal.pone.0306743)
Supplement: S1 Data — (DOCX) [file pone.0306743.s001.docx]

# Supporting information

S1 Data. Analytical data set for the study. (XLSX)

We have made the account of the figshare.com and updated the anonymized data set (also including variable list and measurements) as shown below link:

- Jeon, Junehyung; Woo, Ayoung (2024). Data_PLOS ONE_EMS.xlsx. figshare. Dataset.  [https://doi.org/10.6084/m9.figshare.25348141.v2](https://doi.org/10.6084/m9.figshare.24303208)
